# A Case of Refractory Myxedema Coma

**DOI:** 10.7759/cureus.9737

**Published:** 2020-08-14

**Authors:** Dawn Maldonado, Urja Patel, Nancy Tarlin

**Affiliations:** 1 Internal Medicine, Mount Sinai Medical Center/Elmhurst Hospital Center, New York City, USA; 2 Endocrinology, Mount Sinai Medical Center/Elmhurst Hospital Center, New York City, USA

**Keywords:** myxedema coma, hypothyroidism

## Abstract

We present a case of myxedema coma refractory to traditional treatments. Morbidity and mortality from myxedema coma are frequently due to a missed or delayed diagnosis. It tends to respond very well to intravenous levothyroxine replenishment as long as this treatment is initiated early. We report a case of a 71-year-old man who presented with altered mental status and severe bradycardia who was promptly diagnosed with myxedema coma on laboratory studies sent in the emergency department (thyroid-stimulating hormone 94.74, free T4 0.17, and free T3 0.69). However, while the diagnosis was recognized immediately, and he was treated aggressively with intravenous thyroxine replacement, he strangely remained refractory to treatment for a prolonged period of time. While he did respond to intravenous thyroxine initially, he dramatically decompensated each time he was transitioned to oral therapy. This case brings to question why rarely certain patients fail the transition to oral therapy, and how to treat these patients.

## Introduction

Myxedema coma is a life-threatening emergency resulting from extremely low levels of thyroid hormones [1]. Clinical signs and symptoms include hypothermia, coma, hypotension, arrhythmias, hypercapnea, and hypoxemia. These patients require admission to the intensive care unit, and frequently require intubation.

Fatalities of myxedema coma are caused by severe hypotension, hypoventilation, deterioration of mental status, and renal complications [1]. Even these complications do respond to intravenous thyroid replacement if initiated early [2]. If the diagnosis is recognized early and treated aggressively, and if the patient does not die from the lethal complications while awaiting treatment, then the thyroid function tests rapidly improve within about five days [3].

Cases of refractory hypothyroidism have been described in clinical literature, but no cases of refractory myxedema coma have been described. Multiple studies demonstrate the efficacy of either intravenous levothyroxine or both intravenous levothyroxine and liothyronine, and the patients survive and are able to be transitioned to oral therapy [4]. Subsequent failure of oral therapy is commonly found in the setting of the patient’s non-compliance [5]. Very occasionally, it has found to be in the setting of gastrointestinal malabsorption [6].

We describe a case of myxedema coma that remained refractory to traditional treatment for a prolonged period of time for unclear reasons.

## Case presentation

**Figure 1:**
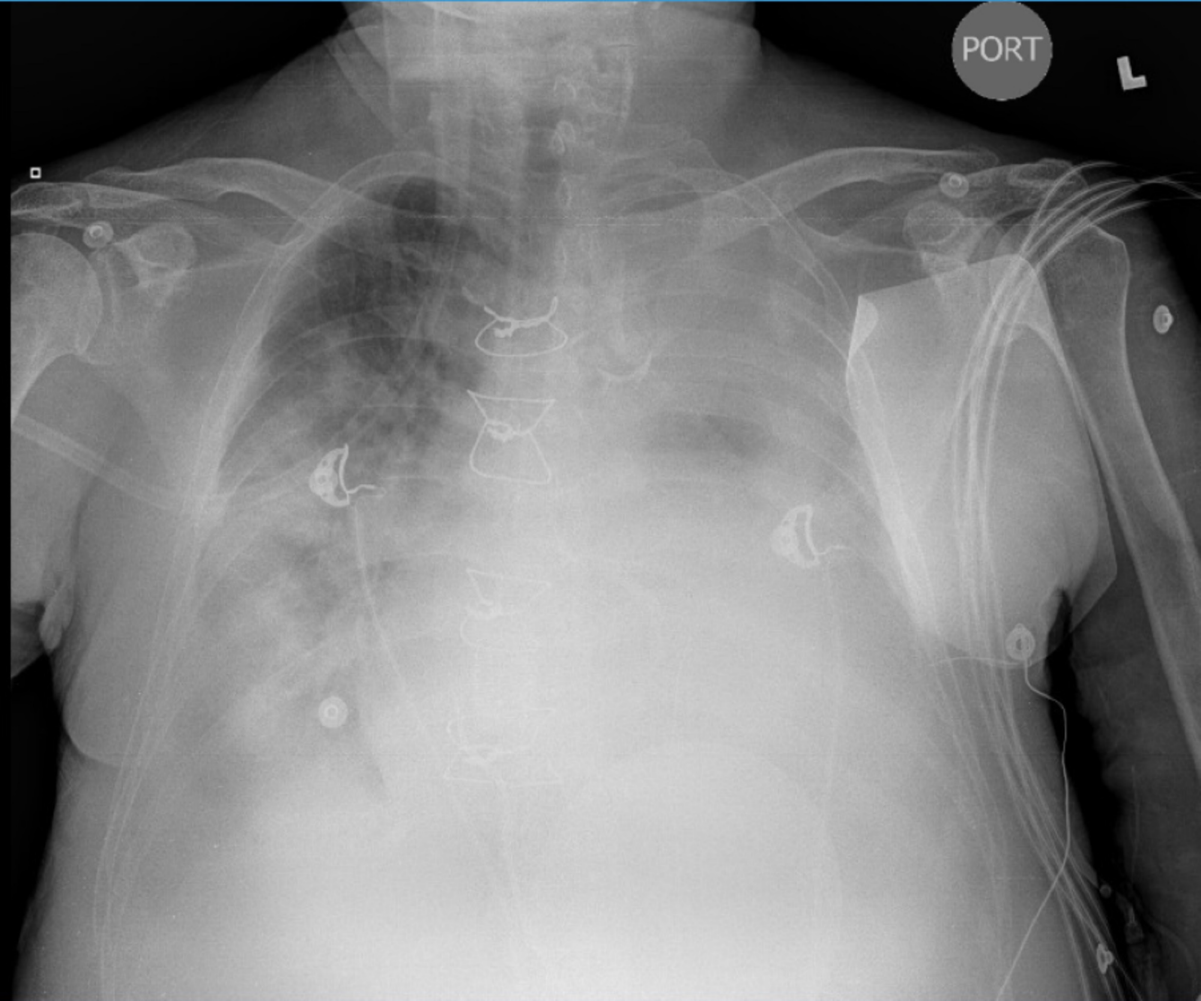
Bilateral Pleural Effusions

We report a case of a 71-year-old man with a history of coronary artery disease, heart failure with reduced ejection fraction, atrial fibrillation, and chronic kidney disease stage 3 who presented with altered mental status and severe bradycardia. His pulse was weak and thready with a rate in the 20s-30s, requiring temporary cardiac pacing. Vitals were notable for severe hypothermia. Imaging demonstrated massive bilateral pleural effusions (Figure 1), and labs demonstrated hypercapneic respiratory failure in addition to renal failure. The differential diagnosis of myxedema coma was promptly recognized in the emergency department. Thyroid function tests were sent and showed thyroid-stimulating hormone (TSH) 94.74, free T4 0.17, and free T3 0.69. Adrenal insufficiency was ruled out with a cosyntropin stimulation test: basal cortisol 15.12, 30-minute cortisol 23, and 1-hour cortisol 23.4.

He was admitted to the intensive care unit and started on stress-dose steroids with intravenous hydrocortisone 50 mg every eight hours and intravenous levothyroxine 100 mcg daily. (The loading dose of levothyroxine 300-500 mcg was held due to his underlying coronary artery disease and atrial fibrillation.) He initially required bilevel positive airway pressure (BiPAP) with low threshold for intubation. Within a day, his free T4 already starting increasing from 0.17 to 0.31, and within eight days it normalized to 1.06. At the same time, his mental status, respiratory failure, and bradycardia resolved, and his renal failure improved. Levothyroxine was transitioned from intravenous to oral 125 mcg daily, and he was downgraded to the floor. However, four days later he became bradycardic again to the 20s with worsening mental status and renal failure, and repeat thyroid function tests again worsened to free T4 0.89.

This pattern in fact happened three separate times where he would improve on intravenous thyroid replacement therapy, but as soon as oral therapy was attempted, he would again decompensate. He even had a cardiac arrest at one point in the setting of this decompensation.

All his medications were reviewed, and it was confirmed that none interacted with levothyroxine absorption: i.e. furosemide, apixaban, carvedilol, atorvastatin, levetiracetam, sevelamer, and basal with sliding-scale insulin. Gastroenterology was consulted due to concern for malabsorption of the oral therapy. He was found to have cholestasis, but this was attributed to his myxedema coma rather than the other way around.

He eventually was very gradually and cautiously transitioned to oral therapy at higher doses (ultimately to 150 mcg daily), and he was discharged to subacute rehab.

## Discussion

We present a case of myxedema coma refractory to traditional treatments. Fatality from myxedema coma is frequently due to a delayed diagnosis, but the condition responds very well to intravenous levothyroxine if initiated early and aggressively [1,2]. However, in our patient the diagnosis was recognized immediately, but strangely remained refractory to treatment for a prolonged period of time.

The patient would improve on intravenous thyroid replacement therapy, but as soon as oral therapy was attempted, he would again decompensate. This phenomenon has been described in the literature, but only in the setting of gastric malabsorption or medication interactions [6]. The following disorders have been described as causes of this gastric malabsorption: lactose maldigestion, celiac disease, small intestine bacterial overgrowth, gastric atrophy, decreased peristalsis, ileus, short bowel syndrome, gastric banding, pancreatic steatorrhea, and cirrhosis.

Bowel wall edema as a consequence of myxedema coma would theoretically be another cause in thyroxine malabsorption [6]. Reports have however consistently demonstrated that this edema fully resolves as long as the intravenous thyroxine successfully increases the free T4 [1,6,7].

None of these conditions were found in our patient why his thyroid function tests would respond appropriately to treatment but then relapse when he was transitioned to oral therapy. Only a few small studies in the literature shed light on what may help in these circumstances. For example, triiodothyronine is absorbed better than thyroxine, and vitamin C has been shown to help with thyroxine absorption; therefore, giving either a combination of thyroxine and triiodothyronine or a combination of thyroxine and vitamin C may be more efficacious in these circumstances than giving thyroxine alone [8,9]. We however only have insufficient data to guide us on how to treat these patients.

## Conclusions

We present a novel case where the diagnosis of myxedema coma was recognized immediately and treated aggressively, where the thyroid function tests responded appropriately and the patient survived the fatal complications, but kept regressing back into myxedema coma when transitioned to oral therapy. Malabsorption could theoretically explain this phenomenon, but this patient did not appear to have any issues with gastrointestinal absorption. He in fact eventually responded well to oral therapy, but only after a full three relapses into myxedema coma.

This brings to question whether our traditional treatments are always effective for myxedema coma. We need to investigate further why rarely certain patients fail the transition to oral therapy, and how to treat these patients.
